# Assessment of salivary pro inflammatory cytokines profile level in patients treated with labial and lingual fixed orthodontic appliances

**DOI:** 10.1371/journal.pone.0249999

**Published:** 2021-04-22

**Authors:** Hosam Ali Baeshen

**Affiliations:** Department of Orthodontics, Faculty of Dentistry, King Abdulaziz University, Jeddah, Saudi Arabia; Harran Üniversitesi: Harran Universitesi, TURKEY

## Abstract

The secretions of certain cytokines, chemokines and growth factors are triggered by orthodontic appliances, which often affect the remodelling of periodontal tissues. Critical cumulative forces are applied by various types of orthodontic appliances to the periodontium. The secretion of such molecules is probably responsible, through molecular and cellular communications, for the optimal resorption of hard tissues in the periodontal setting, which therefore enables the coordination of multiple movements of tooth. This study assessed and compared a wide range of cytokines, cellular marker analysis and defensins present in the saliva samples of human subjected to orthodontic treatment with two different treatment modalities, i.e., conventional lingual and labial fixed orthodontic appliances. A total 40 samples of saliva were obtained, of which 20 were treated with traditional lingual appliances and 20 were treated with labial fixed appliances. After 21 days of treatment, all salivary samples were collected from the subjects. In order to analyse a broad range of soluble cytokine levels in saliva by flow cytometry, a bead-based immunoassay was performed. Cell surface markers were analysed by flow cytometry. Protein levels of saliva for defensins were quantified by ELISA. Non-significant differences were observed in the cytokine levels in the saliva except for the significant effects for CCL2, IL-17A and IL-6. Cellular markers CD45 and CD326 showed high percentage in conventional lingual samples. Defensin levels were found to be lower in conventional lingual patients. Subjects with conventional lingual appliances had significantly higher salivary protein levels of IL-1β, CCL2, IL17A, and IL-6, higher CD45+ and CD326+ cells and lower defensin levels than subjects with fixed labial appliances. The current study provided a clear basis for the development of innovative methods to aid in the improvement of various procedural treatments and orthodontic equipment of next generation.

## Introduction

In common practice, orthodontic treatments utilize different kinds of orthodontic appliances [1–3]. The appliances are described in various types depending on the position in which they are applied for their function [2, 4]. Many differential forces are exerted on the periodontal tissues by all forms of orthodontic appliances [2, 5, 6]. Such mechanical stresses applied on the periodontium allow a wide range of growth factors, cytokines and chemokines to be secreted by the tissue [7, 8]. It has been well known that the secretory proteins in the saliva play a significant indirect role in the induction and resorption of the bone tissue by endogenous signalling pathways, which is also associated in the remodelling of the periodontium complex essential for tooth movement along with periodontal tissues [2, 3, 8, 9]. In order to discern the factors influencing bone formation during treatment, an approachable understanding of the interplay and modifications in the secretion of these proteins is of foremost relevance and the studies so far are seeking to understand the principal molecules and the complicated signal transduction pathways necessary for remodelling the periodontal bone tissues for efficacious orthodontic procedures [8, 9]. A number of cytokines are concurrent and actively secreted in saliva due to orthodontic procedure with distinct appliances, which therefore attribute as molecular signals for bone regeneration and remodelling [3, 6, 10]. Linking the levels of salivary cytokines and defensins within different appliances used for orthodontic treatments has presented practical experience to pursue and continuously achieve greater novel engineered orthodontic appliances that take into account the health of periodontium and aesthetics as well, as there is increasing demand for this [2, 11–13]. We are taking this opportunity in this comparative evaluation to explore a wide range of secreted cytokines and defensins in the saliva as well as salivary cell marker analysis of the patients treated with two different orthodontic appliance modalities.

## Materials and methods

### Sample collections and ethical permissions

As per the institutional ethical committee guidelines, after getting the required informed consent forms from the patients, a total of 40 saliva samples were obtained in sterile tubes, the samples consisted of 20 samples from patients with traditional orthodontic lingual equipment and 20 samples from patients with labial fixed orthodontic appliances. The ethics committee of the King Abdulaziz University approved this study under reference number OD-000030. The saliva samples were diluted two time in phosphate buffer saline (PBS) (Sigma, St. Louis, MO, USA) and further utilized for flowcytometry based cytometric bead analysis for cytokine levels.

### Cytometric bead array for the estimation of cytokine levels in the saliva samples

Cytometric bead array was performed to determine the levels of the cytokines in the saliva. LEGENDplex^™^ Human Essential Immune Response Panel (13-plex) (BioLegend, San Diego, CA, USA) (IL-4, IL-2, CXCL10, IL-1β, TNF-α, CCL2, IL-17A, IL-6, IL-10, IFN-γ, IL-12p70, CXCL8, TGF-β1) was used for the detection of the cytokines. Further experimental protocol was performed according to the manufacturer’s guidelines. Briefly, 25 μL of the saliva sample was incubated with the microbeads for 2 hours. After the incubation, the detection antibodies were introduced subsequently to the tests and incubated for 30 minutes. Further, the samples were washed with wash buffer and centrifuged at 2000 rpm for 5 minutes. The supernatant was removed and the pellet was re-suspended in 200 μL sheath fluid. The samples were then acquired on flow cytometer (Attune NxT, Thermo Fisher Scientific, Waltham, MA, USA) and analysis was performed by using LEGENDplex^™^ Data Analysis Software (BioLegend, San Diego, CA, USA).

### Isolation of cells from saliva and flow cytometry for cell surface marker analysis

The saliva samples were diluted with PBS (1:10) and centrifuged at 2500 rpm for 10 minutes. The pellet obtained was then further subjected to antibody staining and flow cytometry analysis. Briefly, the cells were resuspended in PBS and labelled with anti-CD45-APC and anti-CD326-PE (Miltenyi Biotec, Bergisch Gladbach, Germany). After incubation for 30 minutes at room temperature, the cells were acquired on flow cytometer. 5,000 cell events were acquired in each sample. The percentage was calculated in comparison with the unstained control.

### ELISA for analysis of protein levels of defensins in the saliva samples

HNP-1, HNP-2, HBD-1, and HBD-2 protein levels were analysed using the KRIBIOLISA human ELISA kits (Krishgen Biosystems, Los Angeles, CA, USA). All the saliva samples were diluted 10 times in PBS, and the protocol was performed according to the experimental instructions provided with the kit. The absorbance was read at 450 nm using a spectrophotometer (Multiskan FC, Thermo Scientific, San Jose, CA, USA).

### Statistical analyses

The results were shown as mean ± standard deviation of the values from the three independent experimental values. The data were analyzed by using paired t test (two tailed) on GraphPad Prism 8 software (GraphPad Software, La Jolla, CA, USA) for each cytokine, and the p<0.05 was measured as significant (*p<0.05 and **p<0.01).

## Results

### Elevated secretion levels of CCL2, IL-17A, and IL-6 were observed in patients treated with conventional lingual orthodontic appliances

**Fig 1:**
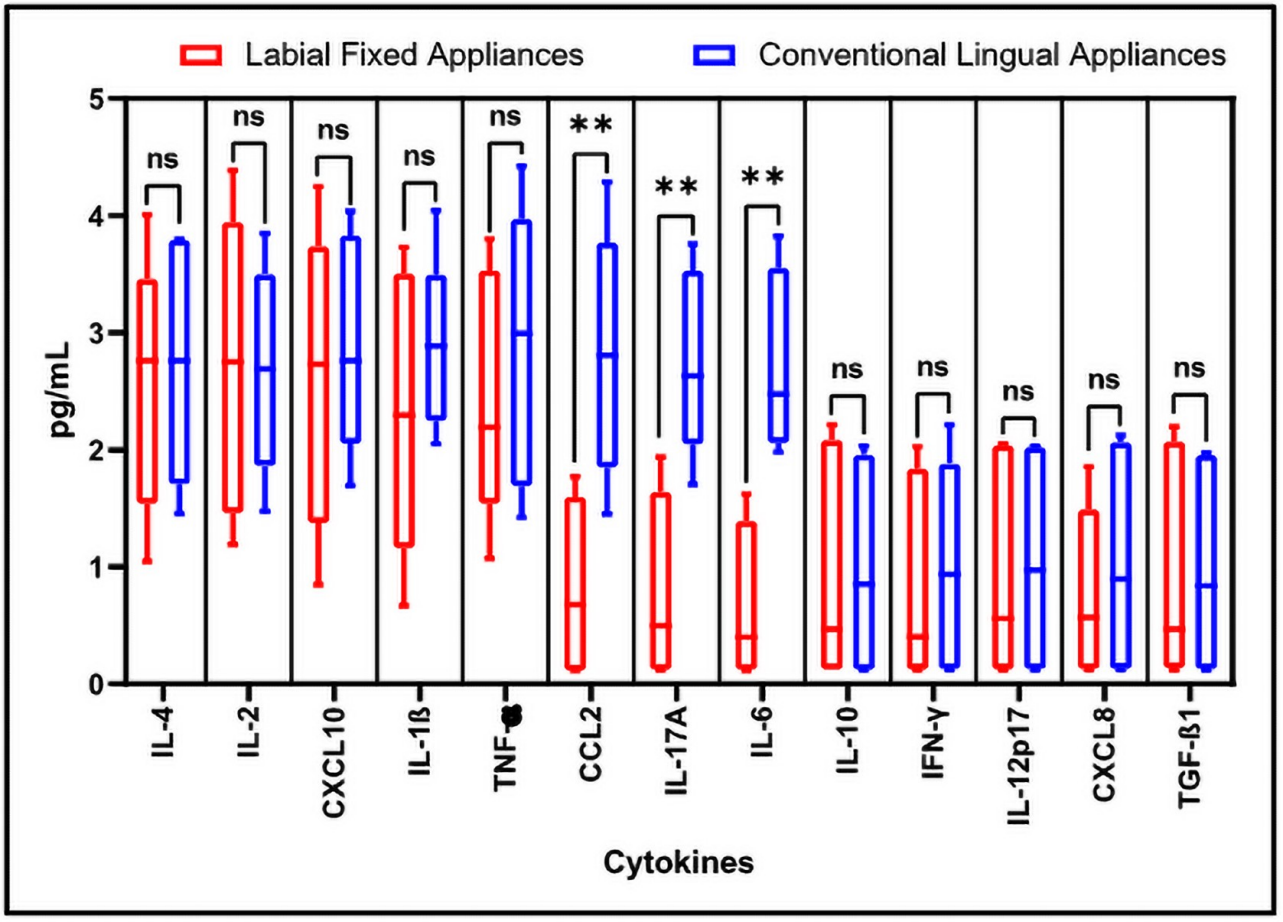
Flow cytometry-based analysis of human cytokines. The quantitation and comparative analysis of cytokines in the salivary samples was assessed by bead-based immunoassay on flow cytometer. Here, ns = non-significant, *p<0.05, **p<0.001. IL-4: Interleukin 4, IL-2: Interleukin 2, CXCL10: C-X-C motif chemokine ligand 10, IL-1β: Interleukin 1 beta, TNF-α: Tumour necrosis factor alpha, CCL2: C-C motif chemokine ligand 2, IL-17A: Interleukin 17A, IL-6: Interleukin 6, IL-10: Interleukin 10, IFN-γ: Interferon gamma, IL-12p70: Interleukin 12, CXCL8: Interleukin 8, TGF-β1: Transforming growth factor beta 1.

Total 13 cytokines including IL-4, IL-2, CXCL10, IL-1β, TNF-α, CCL2, IL-17A, IL-6, IL-10, IFN-γ, IL-12p70, CXCL8, and TGF-β1 were evaluated and compared in the saliva samples of both types of patients (Fig 1). We observed that the cytokines CCL2, IL-17A, and IL-6 were showing significantly higher levels in the saliva from the patients with conventional lingual appliances. The levels of these cytokines were 2 to 4 folds higher in the saliva of patients with conventional lingual treatment than the saliva of patients with labial fixed appliances. Salivary cytokines are biomarkers of periodontal health of individual.

### The percentage of CD45+ and CD326+ cells were more in the saliva of conventional lingual orthodontic appliances

**Fig 2:**
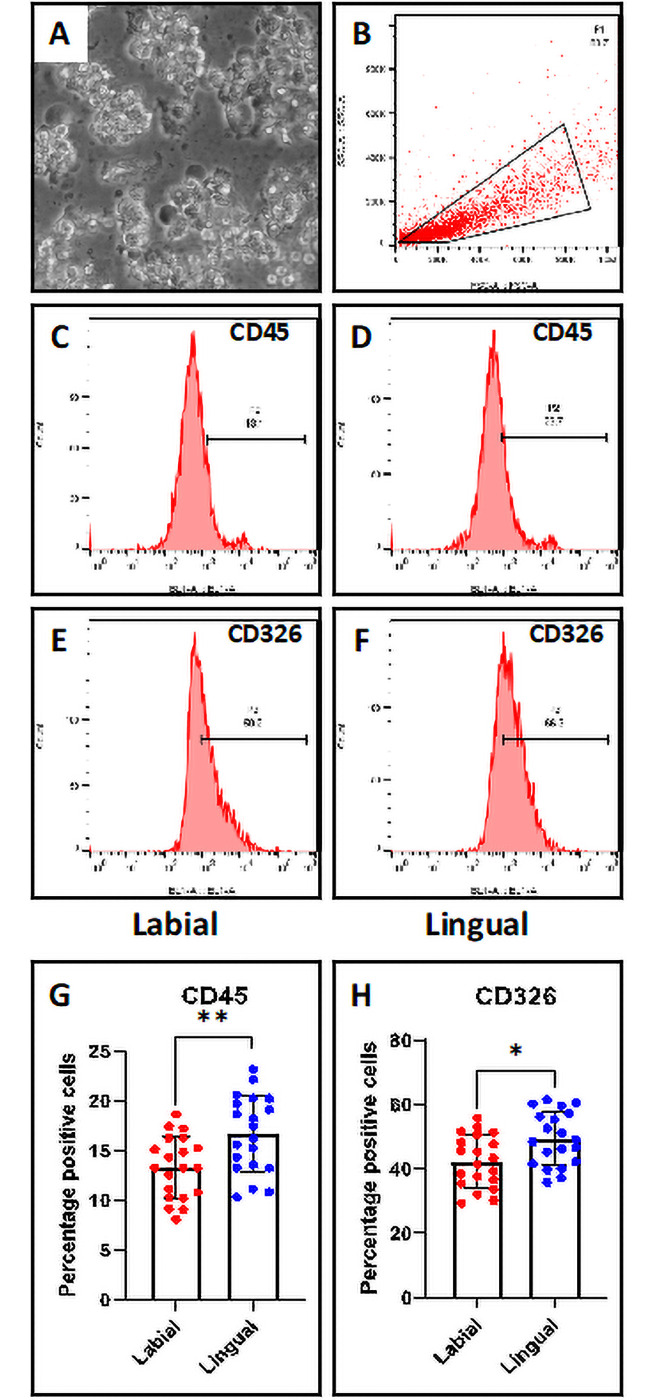
Flow cytometry-based analysis of salivary cells. (A) Salivary cells. Scale bar = 100 μm (B) Gated population of salivary cells. (C & D) CD45 marker analysis of salivary cells from labial and lingual subjects. (E & F) CD326 marker analysis of salivary cells from labial and lingual subjects. (G) Comparative analysis of percentage of CD45+ cells in the saliva from labial and lingual subjects. (H) Comparative analysis of percentage of CD45+ cells in the saliva from labial and lingual subjects. n.s. not significant, *p<0.05, **p<0.001. CD45: Protein tyrosine phosphatase, receptor type, C, CD326: Epithelial cell adhesion molecule.

Salivary cells were subjected to flow cytometric analysis for CD45 and CD326 markers. CD45 is a marker for leukocytes and CD326 (EpCAM) is a marker for epithelial cells. It was observed that the salivary cells from patients with conventional lingual appliances showed higher percentage for both the cell types than those from patients with labial fixed appliances (Fig 2A–2H).

### The levels of defensins HNP-1, HNP-2, HBD-1, and HBD-2 were lower in the saliva of patients with conventional lingual appliances than labial fixed appliances

**Fig 3:**
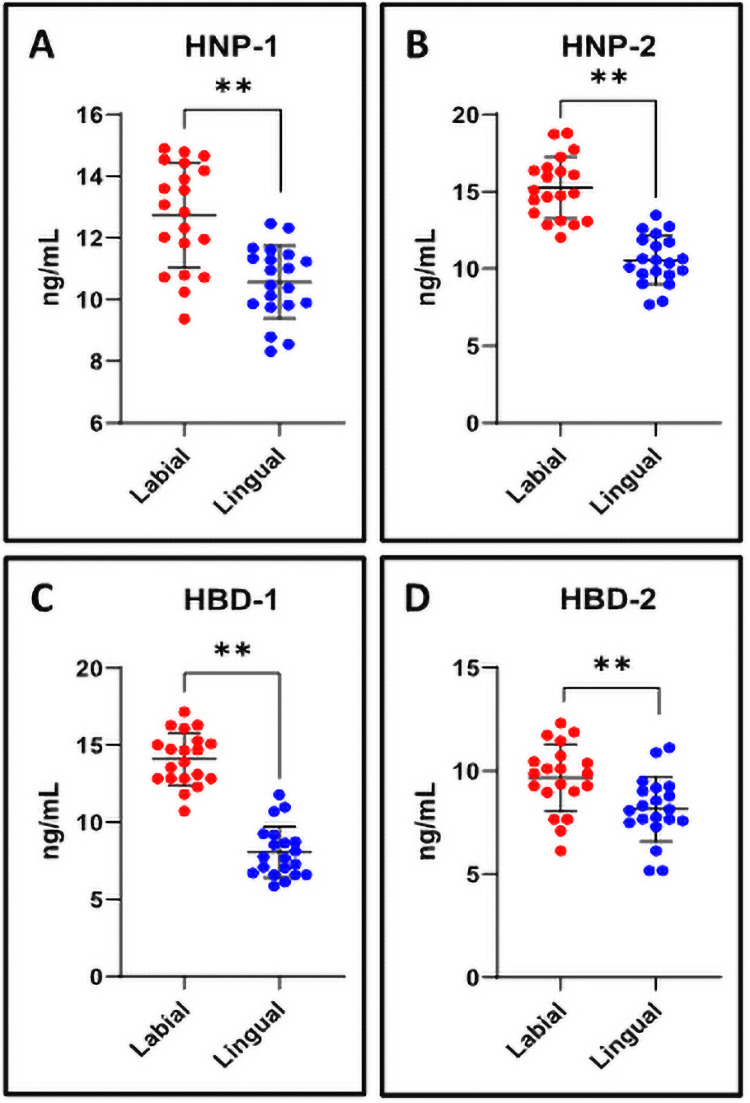
ELISA assay for analysis of human defensins in the saliva. (A-D) The quantitation and comparative analysis of human defensins in the salivary samples was assessed by ELISA on spectrophotometer. n.s. not significant, *p<0.05, **p<0.001. HNP-1: Human alpha defensin 1, HNP-2: Human neutrophil peptide 2, HBD-1: Human beta-defensin 1, HBD-2: Human beta-defensin-2.

ELISA was performed to assess the levels of defensin proteins in the saliva. The saliva of the patients with labial fixed appliances showed higher levels of all four defensins HNP-1, HNP-2, HBD-1, and HBD-2 assessed than that of patients with conventional lingual appliances (Fig 3A–3D).

## Discussion

Patients have to put up with these orthodontic appliances for a considerably long time period [5, 6, 11, 14]. Accordingly, examining the overall characteristics of such appliance-based therapies is critically needed. Although salivary cytokine concentrations are age dependent, disturbed levels of these factors in the saliva indicate poor oral health of individual [15]. It was noticeable that the specific types of appliances have differential influences on the microenvironment that regulates the production of these proteins, which are somewhat accountable for the inflammatory responses involved. Even if only 4 cytokines showed significant differences in both treatment modalities, they may have a substantial impact on future long-term oral health and overall hygiene, along with homeostasis of the multiple oral tissues. Orthodontic appliances often associated frequently with consequences for the incidence and progression of periodontal diseases [16, 17]. It is likely that the observation of these regulatory molecules will necessarily lead to valuable scientific information for the prospects of future orthodontic treatments.

Periodontal diseases usually refer to common inflammatory disorders that trigger innate, inflammatory, and adaptive immune responses [17]. Cytokines are low molecular weight, soluble proteins that are function as chemical messengers for innate and adaptive immunity regulating Innate immunity triggers lymph node to secrete cytokines activating CD4+ lymphocytes. Periodontitis is characterised by the destruction of the tooth-supporting tissues, such as the periodontal ligament [18]. It triggers the local production of pro-inflammatory mediators, production of CCL2 from microorganism chemokine CCL2 also known as MCP-1 creates layer of monocytes, macrophages, and T lymphocytes on the surface of teeth [19]. IL-7 undergoes polarization activates transcription factor ROR-γ. When ROR binds to multiple sites in IL-17A locus along with promotors leads to production of IL-17A induction of many pro inflammatory factors such as TNF-α, IL-6, and IL-1β. IL-17 can play an active role in potentiating innate immunity mechanisms in periodontal environment to increase toll-like receptors responsiveness in human gingival epithelial cells [17, 20]. IL-6 induces T cell proliferation and bone resorption along with regulation of MMP activity. Several studies have inferred that higher levels of IL-6 can have a significant impact on the diagnosis of dental caries and periodontal diseases [16, 18, 21]. Defensins are short anti-microbial peptides that interact with the function and structure of membranes of microbial cells and are present in saliva and other organs. Research accrues that defensins contribute in pathogen resistance and are thought to be part of the innate immune response. Human defensins are commonly distributed in oral tissues including saliva, ducts, salivary glands, and gingival epithelium.

The forces applied on the teeth by the orthodontic appliances produce tissue reactions, which causes remodeling of the alveolar bone [22]. The orthodontic tooth movement depends upon the forces induced on the periodontal ligaments and the alveolar bone remodeling [23–25]. The stresses induced by these orthodontic forces affect the vascularity of PDL resulting in release of various biomarkers such as cytokines, providing a positive environment for bone deposition and resorption [26, 27]. This inflammatory response to orthodontic tooth movement causes release of various cytokines in the GCF. The underlying mechanism of bone metabolism is determined by the levels of various cytokines during orthodontic treatment [28–30]. The initiation, amplification, perpetuation and resolution of inflammatory and immune responses are carried out by cytokines. They are considered as key mediators for tissue damage [31]. An important breakthrough in bone biology was the identification of the role of cytokines in bone remodeling [32].

In order to proceed to a concrete conclusion, additional comprehensive explanation of the biological regulatory networks and of molecular secretion level is required in individuals as well as in more study population. As of now, these findings indicate that the orthodontic treatment with conventional lingual appliances trigger the secretions of salivary cytokines and affect the defensin secretion by increasing the levels of inflammatory cytokines, which can inhibit the oral tissues from continuously repairing and the growth. Nonetheless, we were unable to support these proteins at the gene expression levels. In this research, the exact signalling pathways revealing the synergistic effect along with all the other secreted molecules and cellular components remain unclear. Our study certainly implies that more high-throughput research is needed in this area to improve these essential appliances and also to produce next generation and more efficient orthodontic appliances.

## Conclusion

Comparative study of salivary cytokines in the patients treated with conventional lingual and labial fixed orthodontic appliances indicates that there are some significant differences in the secretions of cytokines in both modes of orthodontic procedures, as demonstrated in a diverse range of cytokines. In the saliva of patients with conventional lingual appliances, the protein levels of IL-1β, CCL2, IL17A, and IL-6 are higher than that of patients with labial fixed appliances. However, with sophisticated experimental techniques, an advanced approach is possible to identify the complex interactions between these factors and regulatory signaling pathways influenced by orthodontic appliances.

## Supporting information

S1 Data (XLSX)
